# Changing Family Relationships During the COVID-19 Pandemic: The Case of Elder Abuse

**DOI:** 10.1093/geroni/igab046.334

**Published:** 2021-12-17

**Authors:** Elsie Yan, Daniel Lai, Vincent Lee

**Affiliations:** 1 The Hong Kong Polytechnic University, Hong Kong, Hong Kong; 2 Hong Kong Baptist University, Hong Kong Baptist University, Hong Kong; 3 The Hong Kong Polytechnic University, The Hong Kong Polytechnic University, Hong Kong

## Abstract

Since the first confirmed case being identified in January 2020, authorities in Hong Kong have implemented various measures in an attempt to control the spread of the disease. These measures include compulsory quarantining of infected persons and those suspected of exposure, temporary closure of high-risk premises, and suspension of public activities and services, encouraging work-from-home arrangement etc. These measures, however, may exacerbate the impact of known risk factors and create new avenues for elder mistreatment. Life stress, financial strains and work-from-home arrangements increase chances of family conflicts, cessation of public services increases burden in the already stressed caregivers. This study examines the changing intergenerational family relations in the midst of the pandemic. A total of 1200 community dwelling senior citizens participated through responding to a telephone survey. Information was collected on participants’ demographic characteristics, perceived disruptions brought about by COVID-19, family relations, physical and mental health, etc. Family conflicts and abuse were commonly reported: 27.8% reported family conflicts, 14.5% psychological abuse, 3.1% physical abuse, 3.9% financial abuse. A large proportion of participants (41.8%), however, also reported improved family relations during the pandemic. Results of logistic regression indicate that advanced age, female gender, poor financial situation were significant predictors for family conflicts and abuse. Contrary to our expectations, pandemic related disruptions in daily lives and perceived safety in the community were not associated in family conflicts and abuse in the present sample.

